# The role of magnetic field in the biopharmaceutical production: Current perspectives

**DOI:** 10.1016/j.btre.2019.e00334

**Published:** 2019-04-04

**Authors:** Alina Rekena, Elina Didrihsone, Kristine Vegere

**Affiliations:** a Rudolfs Cimdins Riga Biomaterials Innovations and Development Centre of RTU, Institute of General Chemical Engineering, Faculty of Materials Science and Applied Chemistry, Riga Technical University, Pulka 3, Riga, LV1007, Latvia; b Bioengineering Laboratory, Latvian State Institute of Wood Chemistry, Dzerbenes 27, Riga, LV1006, Latvia; c Institute of Polymer Materials, Faculty of Materials Science and Applied Chemistry, Riga Technical University, Paula Valdena 3, Riga, LV-1048, Latvia; d Water Research Laboratory, Faculty of Civil Engineering, Riga Technical University, Paula Valdena 1-205, Riga, LV1048, Latvia

**Keywords:** Time-varying magnetic field, Static magnetic field, Magnetic field influence, Mammalian cells, Biopharmaceuticals

## Abstract

Current scientific evidence on the influence of magnetic field on mammalian cell lines used for industrial production of biopharmaceuticals, on human cell lines and on potential cell lines for the biopharmaceutical production is presented in this review. A novel magnetic coupling induced agitation could be the best solution to eliminate sources of contamination in stirred tank bioreactors which is especially important for mammalian cell cultures. Nevertheless, the side effect of magnetically-coupled stirring mechanism is that cells are exposed to the generated magnetic field. The influence of magnetic field on biological systems has been investigated for several decades. The research continues nowadays as well, investigating the influence of various types of magnetic field in a variety of experimental setups. In the context of bioreactors, only the lower frequencies and intensities of the magnetic field are relevant.

## Highlights

• Review on magnetic field influence on industrial mammalian cells for biopharmaceutical production.
• Characteristics of magnetic field in the context of bioreactors.
• Different effects of magnetic field exposure on industrial mammalian cell lines.

## 1 Introduction

The growing interest in biological effects of magnetic field (hereinafter, MF) is connected with an increase of electrical equipment used in everyday life and a fear about possible negative effect on human health [1]. For example, humans are exposed to 50–60 Hz extremely low frequency magnetic field (hereinafter, ELF-MF), which is directly connected to the use, transmission and generation of the electricity [2]. Another aspect of the interest during the last decades is connected with the development of novel technological solutions, such as magnetic drives, for bioreactors [3]. In this construction the drive and driven magnets are connected to the stirrer motor, placed on the end of the stirring shaft and positioned with help of the bearings, and thus the interaction between them is generated [4,5]. A small gap is formed between the shaft and driven magnets with bearings through which the fermentation suspension flows [6]. Depending on the system’s setup, the gap can be called magnetic gap, fluid gap, air gap or bearing gap [6,7].

For the scale of manufacturing stirred tank bioreactors are preferable [8]. However, the conventional agitator shaft used in this type of bioreactors is the most common source of contamination or leakage. Magnetic drive induced stirring system could be the solution of this problem because the stirrer axis does not pierce the vessel [4,5,9].

In case of production of biopharmaceuticals, sterility is a compulsory parameter which can greatly affect the cultivation process. Mammalian cell cultures, which nowadays are the dominant choice for biopharmaceutical manufacturing and generate the majority of revenue, require the highest level of sterility among all types of cultivated microorganisms [4,10,11]. The growing market of biotherapeutics produces the treatment for various hard to win autoimmune, infectious, genetic, hormonal diseases and cancer [12,13].

The side effect of magnetically-coupled stirring mechanism is that cells are exposed to the generated magnetic field. Inside the air gap, cells experience the MF which decreases in strength in the direction to outer vessel wall. Cells are subject to the MF for short periods during the fermentation. The flow speed via the bearing gap depends on the width of the gap and stirrer rotational frequency. In a wider gap, it is higher than in a narrower gap, and in both cases, the liquid flow increases quite linearly with the incensement of the stirrer rotational frequency. For example, liquid flow in the bearing gap in size of 2 mm and 3.5 mm can reach the speed from 5 to 20 mL/s [6]. If fluid flow reaches the speed of 5 ml/s in a vessel with a volume 5 L, it takes around 16.7 min for all fluid to go through the bearing gap once. Consequently, in 1 h’ time a fluid unit resides in the bearing gap subjected to, the maximum MF for 3.6 s. If the flow speed increases two times, the time in the bearing gap increases two-fold as well. However, in case if the vessel volume is increased, the total time for cells in the bearing gap will decrease (if the width of the air gap remains unchanged).

Scientific evidence on the effect of the magnetic field on cell cultures is highly controversial [14]. The aim of this article is to review scientific publications that studied the MF influence on mammalian cell biotechnological properties, such as, growth and apoptosis rates, viability, proliferation, cell cycle distribution, etc.

## 2 Characterization of magnetic field in bioreactor

Throughout the literature, a variety of experimental setups are used to generate MF for cell research. In order to select studies relevant for the scope of this article, characteristics of MF in the context of bioreactors have to be discussed.

**Fig. 1:**
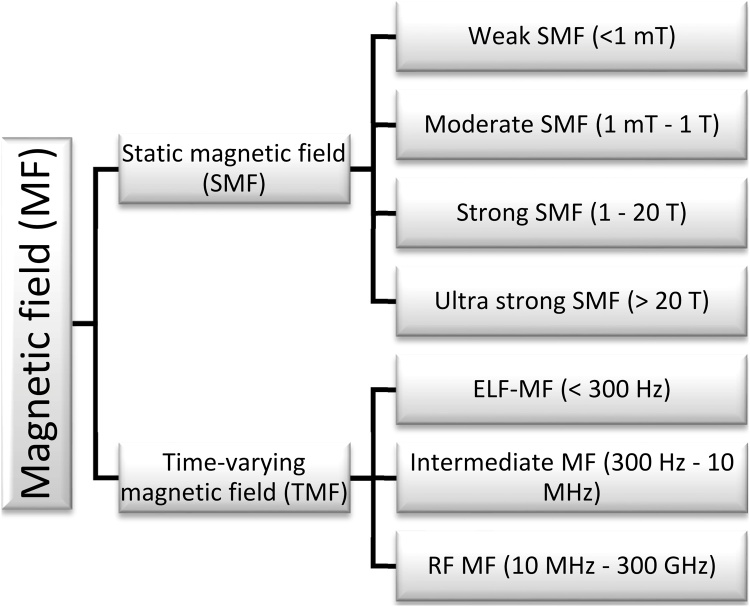
Division of magnetic fields by the type, magnetic flux density and frequency.

From physical perspective, time-varying magnetic fields induce electrical field (Faraday’s law), yielding currents (Ohm’s law) in the conductive media, one like water solutions. Even if these currents usually are considered to be small due to low conductivity and frequencies obtainable in bioreactors, most of authors distinguish between static and time-varying magnetic fields (hereinafter, SMF and TMF, respectively) [15], see Fig. 1.

Less frequently other terms, which explicitly state the time dependency of MF, are used. These are: repetitive [16] or high frequency electromagnetic field [17], continuous and discontinuous electromagnetic field [18], sinusoidal [19], static low level MF [20] etc. In general, magnetic field is called static (SMF) if its intensity or direction over time does not change [15,21,22]. MF can be generated either electrically using direct current or mechanically using permanent magnets. MF with changing intensity or direction over time has a common term time-varying MF. It is commonly used to describe pulsed [23,24] and alternating MFs [25]. TMF can be electrically generated using alternating current or mechanically generated by rotating permanent magnets.

In magnetically-coupled stirring systems MF is usually mechanically generated. Although (quasi-) periodical in time and space domains, these fields usually are not harmonic, thus can be called pulsed. Fourier spectrum of components of pulsed fields (as of any pulsed function) exhibits an infinite number of harmonics with a domination of few base frequencies [26].

The strength of MF within the air gap induced by magnetic couplings depends on a number of factors, including the materials used, the shape, width and height of permanent magnets, the number of pole pairs, the axial length and the air gap [27]. The smaller is the air gap, the higher magnetic flux density and vice versa [[27], [28], [29]].

According to the results of numerical modelling for static magnetic field (thus, an impact of eddy currents in the reactor walls are neglected), the magnetically-coupled stirring mechanisms can produce maximum magnetic flux density of 0.87 T, 1.36 T and 1.02 T at a distance of not less than 2 mm (the size of the air gap) in bioreactors of 1 m^3^, 4 m^3^ and 15 m^3^ size, respectively [27]. The lowest frequency of a pulsed field can be calculated by multiplying the revolutions per second of the rotor with the number of pole pairs for magnetic coupling. According to our estimations, it will be well below 1 kHz. The test bench in one of the studies consists of an electric motor with rotational speed up to 3000 rpm (or 50 rps) and magnetic coupler [27]. The number of pole pairs should be 5 or 6 for bioreactors with volumes 1 m^3^ and 4 m^3^, and 15 m^3^, respectively [27]. This gives base frequency up to 300 Hz. In mammalian cell cultures, however, the rotational speeds are in general lower – ranging from over 500 rpm in small laboratory bioreactors to approximately 100 rpm in very large fermenters [30]. Therefore, MF base frequency would barely reach 50–100 Hz. During the rotation of magnetic coupling stirrer, the actual MF on the cells depend on the movement of cell suspension inside the air gap. The MF frequency on the cell suspension layers close to the drive magnet has a maximum value which then decreases to 0 Hz for cells in suspension layers located at the driven magnet.

In present article, we review research papers where mammalian cell cultures relevant for biopharmaceutical production have been studied under the MF below 1.5 T magnetic flux density and 1000 Hz frequency. Although, cell cultures of our interest have been also studied in MFs of higher intensities and frequencies [17,[31], [32], [33], [34], [35], [36]], these papers are not further discussed. The article was prepared using data bases Scopus, Web of Science, Google Scholar, Science Direct and includes references for the period 1995−2018. The number of research papers studying the effects of ELF-MF on biological systems dominate over the articles of other types of MF [37].

## 3 Magnetic field influence on mammalian cells

Advantages of mammalian expression systems for the biotherapeutic protein production, have been widely reviewed in the scientific literature. These are: the ability to produce large, complex proteins, to provide improved stability and reduced immunogenicity, to secrete proteins, to adapt to various cultivation process parameters, etc. [10,[38], [39], [40], [41], [42], [43], [44]]. Many different mammalian cell lines have been subjected to various MFs for the research purposes [14,45]. Only those being mentioned with regard to recombinant protein expression are reviewed here. These are: Chinese hamster ovary (hereinafter, CHO) cells, murine myeloma cells (NS0 and Sp2/0), baby hamster kidney (BHK21) as being typical biopharmaceutical host cell lines measured per number of products in the market [12,46,47]. A separate subsection is devoted to the human cell lines that are important for production purposes. The shift in the industry toward the use of human cell lines has been discussed by several review articles recently [[47], [48], [49], [50]]. As well as, other mammalian cell lines that have been mentioned in the context of recombinant protein expression even on a laboratory scale, are reviewed. Mammalian cell lines closely related to those selected for a review here, such as murine thymocytes, 3DO, U937, HeLa, FRTL-5, K562, SH-SY5Y, U87, RaJi, HaCat, HL60, have also been subjected to various MFs [14,45].

### 3.1 Industrial biopharmaceutical cell lines

The main host for production of therapeutic proteins is an epithelial-like CHO cell line. Great adaptability and ease of maintenance have let them to become the mammalian equivalent of the model bacterium *E. coli* [51,52]. Compared to other cells of interest for this review, CHO cell line has been studied the most. The reported results are controversial, especially, for TMF exposure. Miyakoshi et al. [53] used 50 Hz, 5 and 400 mT ELF-MF with exposure time of 24 h for investigation of the neuron derived orphan receptor (NOR-1) gene mRNA expression. Results showed that only 400 mT ELF-MF increased NOR-1 mRNA levels up to 6 h of the exposure, afterwards decreasing to control levels. As well as Restrepo et al. [16] investigated 50 Hz, 40 min ELF-EMF effect on CHO cells, changing the magnetic flux density from 0.4, 1.4, 2.13, 1.49 and 2.53 mT. Results in all variations showed increased cell proliferation rate. Walleczek et al. [54] and Miyakoshi et al. [55] investigated 60 Hz ELF-MF effect on the mutation frequency with magnetic flux density of 0.7 mT and 5 mT, respectively. Both experiments showed no effect on mutation frequency. Ding et al. [56] examined micronuclei formation under 60 Hz, 5 mT, 24 h ELF-MF. Ding et al. found no changes in micronuclei frequency.

Considerably less information was available for the effects of SMF on the CHO cell line. Nakahara et al. [57] investigated effect of SMF of 1 T for 18 h. Experiments showed no effect on cell cycle distribution. In addition, Nakahara et al. reported no effect on micronuclei frequency or on cell growth.

NS0 are murine myeloma (plasma tumor) suspension cells originally created from immunoglobulin-producing murine plasma-cell neoplasms (plasmacytomas) and cloned so that they do not anymore secrete immunoglobulin (Non Secreting is abbreviated as NS) [58]. Sp2/0 is a hybridoma cell line originated from the fusion of the murine myeloma cell line of the same origin as of NS0 with mice spleen cells [59]. BHK21 is a fibroblast-like adherent cell line originally derived from 1-day-old Syrian hamster kidneys [60]. These cell lines, however, were not studied under MF exposure at low frequencies.

### 3.2 Human cell lines

The main advantage of human cells is reduced immunogenicity of proteins that they synthesize [47,50]. Although, human cell lines are usually employed for the research purposes, several of them are exploited for the production of licensed protein therapeutics. Many cell lines in this section are a result of an in-house research and development and protected under the intellectual property rights of biopharmaceutical companies.

HEK-293 is a long-ago established cell line with several derivative versions also widely used in the scientific research. Cells were isolated from normal human embryonic kidneys and show epithelial character [61]. Some neuronal properties of this cell line have been reported [62]. Commercially, this is the most widely used human cell line by various companies. Recombinant coagulation factors VIII and IX (FVIII, FIX) and drotrecogin alfa are being produced in these cells [47,63]. The effect of MF on HEK-293 has been observed by Fan et al. [64], who investigated pulse 7 Hz, 6–25 mT MF exposure on calcium ion current profile. Results showed earlier appearance of ion channel in opening, earlier reach of the whole cell current maximum, and earlier return back to the zero of the current. However, after the pulsed MF exposure was stopped, all processes returned to the original appearance. Cui et al. [65] exposed HEK-293 cells to 50 Hz, 0.2 mT ELF-EMF for 1 h. Observations showed inhibition of T-type calcium channels via specific signaling pathway. However El-Gaddar et al. [66] investigated 0.5 T SMF effect, and exposing cells for 72 h did not show any changes on electrical properties, growth, and morphology.

HKB-11 is another hybrid cell line derived from non-tumor human embryonic kidney (HEK) and human suspension B cells with an aim to reduce cell aggregation properties [67]. Successful overexpression of recombinant proteins, including coagulation factor VIII has been demonstrated [[68], [69], [70]]. This cell line is patented by Bayer HealthCare and commercial production using this cell line is under development [63]. However, no studies on MF influence could be found.

HT-1080 cells were isolated from a fibrosarcoma (tumor of the fibrous tissue of the bone) patient in 1972. Phenotypically, HT-1080 are rounded tumor cells [71]. Nowadays, several commercial products using this cell line are manufactured by Shire, Inc. In contrast to other approaches, their technology is not based on DNA recombination, but targeted activation of an endogenous gene [72]. Chen et al. [73] investigated 1 mT EMF exposure for 48 h, and observed increased apoptosis rate. Static low level MF of 0.2–2 μT on HT1080 after 6, 12 and 24 h showed decreased ROS activity [20].

Several other human cell lines show a great potential for biopharmaceutical production. For example, PER.C6 cell line was originally created from human embryonic retina (HER) cells [74], but as a platform for recombinant protein production it is marketed by Crucell Holland BV. Successful expression of recombinant therapeutical monoclonal antibodies [75,76] and alpha-1-antitrypsin (abbreviated as A1PI, A1AT, or AAT) has been reported [77]. Also, CAP and its derivative cell line CAP-T, originally derived from human amniocytes of non-tumor origin [78,79] are currently marketed as a platform for biopharmaceutical research by CEVEC Pharmaceuticals. Phenotypically, amniocytes can be fibroblast-like, epithelial-like and amniotic fluid (AF) cells, and it is not yet clear to which phenotype CAP cells correspond [80,81]. Successful overexpression of human AAT has been demonstrated [79]. AGE1.HN is a cell line developed from human brain tissue. Cells have been transformed for recombinant protein production and have neural lineage cell properties [82]. However, AGE1.HN lack the expression of glial markers. This cell line is licensed by Probiogen AG. Expression of recombinant A1AT using this cell line has been reported [83,84]. RS are normal renal proximal tubular cells isolated from human kidneys. This cell line demonstrates typical epithelial morphology. It has been engineered to overexpress recombinant human erythropoietin [85]. However, no studies on MF influence on these cell lines have been reported.

### 3.3 Cell lines with a potential for biopharmaceutical production

Cell lines that are selected for this section have been successfully tested on a laboratory scale as expression systems for recombinant protein production.

TE671 cell line was isolated from a cerebellar medulloblastoma (brain tumor) patient. It is a neuronal cell line, but the properties of rhabdomysarcoma have also been detected. Phenotypically, the TE671 cell line demonstrates an epithelial, adherent spindle-shaped morphology and stable transfection to overexpress A1AT has been demonstrated [86]. Ho et al. [87] have investigated effect of 3 mT SMF on TE671 cell line with exposure time of 72 h. Results showed no effect on cell viability.

HuH-7 is a long-ago established cell line isolated from hepatocellular carcinoma (liver tumor) patient in 1982. These adherent cells demonstrate epithelial morphology with pavement-like arrangement [88]. Despite some changes in morphology compared to the normal cells, NuH-7 have maintained liver cell functions as shown by the production of plasma proteins [89]. Expression of recombinant factor IX (for hemophilia treatment) [90,91] and human erythropoietin (EPO) [92] has been reported. This cell line has not been studied under the influence of the MF.

Production of recombinant clotting factors has been attempted using several other tumor cell lines derived from liver. This is a special occasion because coagulation factors are naturally synthesized in human liver. The following cell lines have been used for recombinant protein production: mesenchymal stem cell-like SK-Hep-1, epithelial hepatoblastoma Hep-G2 [70,[93], [94], [95], [96], [97], [98]]. These cell lines have been studied under the influence of the magnetic field. MF influence on SK-Hep-1 cell line is reported by Huang et al. [19], who investigated influence of 50 Hz, 20 mT sinusoidal MF with exposure time up to 4 days. Results showed decreased cell proliferation, increased osmolarity and enhanced extracellular Na^+^, K^+^ ion concentrations. Cid et al. [99] observed increased cell proliferation by exposing HEP-G2 to 50 Hz, 10 μT ELF-MF for 4, 5 and 7 days. Chen et al. [100] observed no effect on apoptosis, cell proliferation and calcium levels by exposing HepG2 cells to 0.5 T SMF.

COS is an adherent fibroblast-like cell line, originally derived from a normal African green monkey kidney cell line CV-1, which also has been engineered for recombinant protein production [101,102]. This cell line, however, has not been mentioned with regard to MF studies. A non-tumor epithelial cell line Vero, established from African green monkey kidneys [103], has been studied after MF exposure also at low frequencies. MF effect on Vero cells was reported by Mihai et al. [18], investigating continuous and discontinuous 100 Hz, 5.6 mT EMF exposure for 45 min. Effect was investigated 48 h after the exposure and it was revealed that the EMF exposure increased DNA damage, the tail length and quantity of DNA in tail. SMF influence was observed by Buemi et al. [104], investigating 0.5 mT SMF exposure on Vero cells for 6 days. Results showed reduced proliferation rate and apoptosis, and increased cell count with necrotic morphology. Summary of the reported effects of magnetic field on the selected cell lines is listed in Table 1.

**Table 1:** Summary of magnetic field influence on mammalian cell lines.

Cell line	MF applied	Frequency	Magnetic flux density	Exposure time	Exposure effect	Reference
CHO-K1	ELF-MF	50 Hz	5 mT	24 h	No effect on neuron derived orphan receptor (NOR-1) gene mRNA expression	[53]
			400 mT	24 h	Increased (NOR-1) mRNA levels up to 6 h of exposure, decreased afterwards to control level at 24 h	
CHO-K1	ELF-EMF	50 Hz	0.4 mT, 1.4 mT, 2.13 mT, 2.49 mT and 2.53 mT	40 min	Increased cellular proliferation rate	[16]
CHO-K1	Repetitive EMF	50 Hz	2.49 mT	40 min per 12 h for 4 days	Increased cellular proliferation rate	[16]
CHO	ELF-MF	60 Hz	0.7 mT	12 h	No effect on mutation frequency and radiation-induced cytotoxicity	[54]
CHO-K1	ELF-MF	60 Hz	5 mT	24 h	No effect on bi-nucleated cell frequency carrying micronuclei	[56]
CHO-K1	ELF-MF	60 Hz	5 mT	6 weeks	No effect on mutation frequency	[55]
CHO-K1	SMF	–	1 T	18 h	No effect on micronucleus frequency	[57]
HEK293	Pulse MF	7 Hz	6 – 25 mT	90 sweeps	Reversible changes on Calcium ion current profile	[64]
HEK293	ELF-EMF	50 Hz	0.2 mT	1 h	Inhibition of native T-type calcium channels through AA (arachidonic acid)/LTE4 (leukotriene E4) signalling pathway	[65]
HEK293	SMF	–	0.5 T	72 h	No effect on electrical properties, growth, and morphology	[66]
HT1080	EMF	No info	1 mT	48 h	Increased apoptosis rate	[73]
HT1080	Static low level MF	–	0.2 – 2 μT	6 h, 12 h, 24 h	Decreased ROS (reactive oxygen species) activity	[20]
TE671	SMF	–	0.003 T	72 h	No effect on cell viability	[87]
SK-Hep-1	Sinusoidal MF	50 Hz	20 mT	1, 2, 3, 4 days	Decreased proliferation; enhanced extracellular Na−+, K−+ ion concentrations; increased osmolarity	[19]
HepG2	ELF-MF	50 Hz	10 μT	4, 5, 7 days	Increased cell proliferation	[99]
HepG2	SMF	–	0.5 T	72 h	No effect on cell proliferation, did not influence the calcium level significantly, no increase of apoptosis	[100]
Vero	Continuous and discontinuous EMF	100 Hz	5.6 mT	45 min	Increased DNA damage, the tail length and quantity of DNA in tail for both cEMF and dcEMF (effect evaluated 48 h after exposure)	[18]
Vero	SMF	–	0.5 mT	6 days	Reduced proliferation and apoptosis, increased necrosis	[104]

## 4 Conclusions

In present article, we narrowed down the scope of research to the specific mammalian cell lines that are relevant for the biopharmaceutical production processes representing a rapidly rising market segment of pharmaceuticals worldwide. As well as, only the lower magnetic flux densities and base frequencies (under 1.5 T and 1 kHz) were considered relevant in the context of bioreactor processes. The amount of research papers in this field is limited. The current industrial cell cultures have been studied the most, whilst the human cell lines now entering the market for industrial production are not researched under MF exposure at all. However, cell lines with the potential for therapeutic protein expression have been quite extensively studied in most cases due to the fact that these cells are mainly associated with the cancer research rather than biopharmaceutical production. From the selected mammalian cell lines, CHO, HEK293, HT1080, TE671, SK-Hep-1, HepG2 and Vero have been studied under the magnetic field influence at low frequencies. The majority of other cell lines under the scope of this study are a trademark or trade secret of companies that have invested their time and money to modify them to serve the purpose of therapeutic protein production. Moreover, these companies have no interest in the study of MF effect themselves, and obviously they would not want to share the cell line with competitors for this purpose. Cell characteristics, studied by the reviewed papers, include cell proliferation and viability, mutation frequency, micronuclei formation and ion channel activity, as well as, (NOR-1) mRNA levels, and DNA damage. Overall, studies using a variety of experimental setups are difficult to compare.

For CHO cells the MF influence is neutral with regard to the cell growth rate, cell cycle distribution and mutation frequency, micronuclei formation and even positive with regard to proliferation. Especially, the effects are all over neutral for the SMF exposure. Increased gene expression at the beginning of TMF exposure have been reported.

With regard to human cell lines, SMF exposure had no change on HEK293 cell electrical properties, growth, and morphology. However, an inhibition of calcium channels, earlier appearance in ion channel in opening and modifications in the profile of the whole cell calcium channel current as a result of TMF exposure have been reported. With regard to Shire’s HT-1080 cells, only negative effects have been reported. Accordingly, increased apoptosis as a result of TMF exposure, as well as, decreased ROS activity due to SMF exposure have been reported.

Also, a negative, inconsistent influence of the TMF is shown on liver tumor cell lines SK-Hep-1 and Hep-G2. Increased and decreased proliferation, enhancement in extracellular ion concentrations were reported. At the same time, again no effect of SMF exposure on cell apoptosis, proliferation and calcium levels has been reported. Similarly, no effect on cell viability for TE671 cells from the SMF exposure has been reported. For Vero cells, however, it does not hold true, as there is reduced proliferation and apoptosis, increased cell count with necrotic morphology from the SMF exposure. Also, increased DNA damage as a result of TMF exposure has been reported.

In this article we provide the perspective on MFs present in a bioreactor during industrial production processes and review the MF influence on cells at those frequencies. In general, no effect from the available studies of MF exposure on growth and viability of industrial biopharmaceutical cell cultures has been reported. For other groups, highly inconsistent results on growth rate and proliferation have been reported. Yet, so many cell cultures have not ever been studied under the MF exposure, especially the cell lines of human origin under the proprietary development of biotechnology companies. Overall, we believe that the information summarized in this article will not only help increase awareness of the developments in both cellular and technological systems for the biopharmaceutical production but will also clear the bias of negativity with which the MF exposure on biological systems is often perceived.

Based on the reviewed data in this study, we cannot observe any correlation between the MF frequency and magnetic flux density and the presence or absence of the effects on the cells. Also, a number of experiments using MF with the same characteristics or investigating the same effects on cells are too low to make an objective comparison. In a wider context, the perception of negative effects from the TMF is probably too exaggerated. We observed that most of the experimental setup in the reviewed articles was developed in such way that cells were placed for exposure right next to, right under or in between the MF generator, which would never be true for humans and the electrical appliances. This is also verified by the physics that by increasing the distance, the MF reduces [29] and, therefore, the induced effect reduces or vanishes.

## Conflict of interest

All authors declare that there was no conflict of interest
